# Health literacy and psychological well-being in community-dwelling older people: data from FelizIDADE project

**DOI:** 10.1192/j.eurpsy.2025.1739

**Published:** 2025-08-26

**Authors:** S. Lima, S. Martins, J. L. Martins, R. Carvalho, H. Correia

**Affiliations:** 1 Innovation in Health and Well-Being Research Unit (iHealth4Well-being), CESPU, Porto; 2 PrestigeHealth; 3 Centro Social de Requião, Vila Nova de Famalicão, Portugal; 4 Social Work, Centro Social de Requião, Vila Nova de Famalicão, Portugal

## Abstract

**Introduction:**

Health literacy (HL) is characterised as the ability to understand health and involves people’s knowledge, motivation and skills to access, recognise, evaluate and apply health-related information. It is an important topic in the context of healthcare, with studies suggesting that low levels of HL are predictors of adverse health outcomes. Socioeconomic status, age, race, cognition and level of education are factors that contribute to HL levels, with older age being strongly associated with poor HL. However, the relationship between HL and the psychological well-being of older people has not been explored as much in the literature.

**Objectives:**

To describe HL and psychological well-being levels in community-dwelling older people and to analyse the association between these two constructs.

**Methods:**

The data analysed in this study derives from a longitudinal research project called FelizIDADE, whose main objective was to empower older people to promote health and HL. A sample with older people aged 65 years and over, living in the municipality of Vila Nova de Famalicão, in Portugal was included. All participants were assessed with a comprehensive research protocol, which comprised, amongst others, the European Health Literacy Questionnaire (HLS-EU-Q16) and the Psychological Well-Being Scale (PWBS). For statistical analysis, non-parametric tests were used, since data did not follow a normal distribution.

**Results:**

A sample with 59 community-dwelling older people was considered, with a mean age of 72 years (SD=4.5). The majority was female (59%), married (83%) and 91.5% had the completed primary school. Around 83% lived with their spouse and 88% were retired due to age. Statistically positive correlations were identified between the HLS-EU-Q16, age (rs=0.0274; p<0.05) and PWBS (rs=0.336; p<0.01). The total of HLS-EU-Q16 also correlated positively with the total of four domains of the PWBS: Autonomy (rs=0.412;p<0.01); Personal Growth (rs=0.280; p<0.05); Positive relations with others (rs=0.275; p<0.05) and Purpose in life (rs= 0.379; p<0.01).

**Conclusions:**

It is well known that HL plays a fundamental role in older people and lower levels are related to negative impacts on health. This reinforces the importance of understanding the factors that are associated with poor HL. The present findings provide empirical insights into the association between HL and psychological well-being among older people living in the community. Furthermore, these results emphasise HL as an individual resource in promoting well-being.

**Disclosure of Interest:**

None Declared

